# Epidemiology and comorbidities in idiopathic pulmonary fibrosis: a nationwide cohort study

**DOI:** 10.1186/s12890-023-02340-8

**Published:** 2023-02-04

**Authors:** Jang Ho Lee, Hyung Jun Park, Seonok Kim, Ye-Jee Kim, Ho Cheol Kim

**Affiliations:** 1 Division of Pulmonology and Critical Care Medicine, Department of Internal Medicine, Asan Medical Centre, University of Ulsan College of Medicine, 88 Olympic-Ro 43-Gil, Songpa-Gu, Seoul, 05505 Republic of Korea; 2 Department of Clinical Epidemiology and Biostatistics, Asan Medical Centre, University of Ulsan College of Medicine, Seoul, Republic of Korea

**Keywords:** Comorbidity, Idiopathic pulmonary fibrosis, Incidence, Pirfenidone, Prevalence

## Abstract

**Background:**

Idiopathic pulmonary fibrosis (IPF) is frequently accompanied by comorbidities, with the management of these comorbidities crucial for clinical outcomes. This study investigated the prevalence, incidence, changes over time, and clinical impact of comorbidities in IPF patients, based on nationwide claims data in South Korea.

**Methods:**

This retrospective cohort study utilised nationwide health claim data in South Korea between 2011 and 2019. Patients with IPF were defined as those with ICD-10 code J84.1 and Rare Intractable Disease code V236 who made at least one claim per year. Patients were classified by sex, age, pirfenidone use and burden of comorbidities, and differences among groups were determined.

**Results:**

The yearly prevalence rate of IPF increased from 7.50 to 23.20 per 100,000 people, and the yearly incidence rate increased from 3.56 to 7.91 per 100,000 person-years over time. The most common respiratory comorbidity was chronic obstructive pulmonary disease (37.34%), followed by lung cancer (3.34%), whereas the most common non-respiratory comorbidities were gastro-oesophageal reflux disease (70.83%), dyslipidaemia (62.93%) and hypertension (59.04%). The proportion of some comorbidities differed by sex, age and use of pirfenidone. The proportion of lung cancer was higher in patients treated with pirfenidone, whereas the proportion of anxiety and depression were lower in patients not treated with pirfenidone. Charlson comorbidity index ≥ 4 was associated with increases in hospitalisations and total medical costs.

**Conclusions:**

The yearly prevalence and incidence of IPF and comorbidities in Korea increased over time. These comorbidities affected the use of pirfenidone and medical resources.

**Supplementary Information:**

The online version contains supplementary material available at 10.1186/s12890-023-02340-8.

## Background

Idiopathic pulmonary fibrosis (IPF), defined as a chronic, progressive, fibrotic lung disease of unknown causes, is the most common type of interstitial lung disease [1]. Although the annual incidence of IPF has been reported to range from 0.9 to 13.0 per 100,000 persons worldwide, its clinical course is debilitating, with a median survival of 3–5 years after diagnosis, and it has substantial socio-economic burden [2–4].

Treatment options for patients with IPF are often limited, despite the use of antifibrotic agents such as pirfenidone and nintedaninb [5, 6]. Because IPF occurs mainly in elderly patients, many of whom have comorbidities, management of these comorbidities is crucial for their clinical outcomes [7]. Chronic obstructive pulmonary disease (COPD), lung cancer, pulmonary embolism and pulmonary hypertension are well-known respiratory comorbidities [8]. Non-respiratory comorbidities include gastro-oesophageal reflux disease (GERD), cardiovascular disease and psychiatric diseases [8]. Several previous studies have assessed the prevalence and clinical impact of comorbidities in patients with IPF [8, 9]. An increased burden of comorbidities, especially lung cancer and cardiac disease, is associated with increased mortality [10]. In these previous studies, however, the study population was based on a single centre or cohort design, focused on individual comorbidities rather than each patient’s entire comorbidity burden, or did not consider changes in comorbidities over time or treatment with antifibrotics [11–13]. The present study utilised nationwide claims data to investigate the prevalence and incidence of comorbidities, their changes over time and their clinical impact on patients with IPF in South Korea.

## Methods

### Data source and study population

This was a retrospective cohort study based on nationwide health claims data in South Korea. De-identified nationwide health claims data between 2011 and 2019 were collected from the database of the Korean Health Insurance Review and Assessment (HIRA), which includes all health insurance claims from the Korean National Health Insurance System (NHIS) and therefore covers most of the population. The HIRA database presents all information on healthcare utilisation by outpatients and inpatients, including their demographic characteristics, diagnoses, and prescribed medications. Diagnostic codes were determined using the 10th revision of the International Classification of Disease and Related Health Problems (ICD-10). The study was approved by the Institutional Review Board of Asan Medical Center (No. 2020-1000), which waived the requirement for informed consent due to the retrospective nature of the study. The study was performed in accordance with the principles of the Declaration of Helsinki.

Patients in the HIRA database were defined as having IPF if they had made at least one claim per year under the ICD-10 code J84.1 of the medical care system. Because patients in South Korea with confirmed IPF were enrolled as the Rare Intractable Diseases (RID), we additionally used RID code V236 to define patients with IPF patients. In South Korea, patients assigned RID code V236 can benefit from reduced medical costs from the NHIS. Hospitals charge the NHIS for the remainder, but if their diagnosis does not meet RID diagnostic criteria, the NHIS refuses to pay these hospital costs. This study therefore assumed that patients were accurately diagnosed at the time of RID registration. Therefore, IPF in the present study was defined as a composite of ICD-10 code J84.1 and RID code V236. The prevalence was estimated by including patients who had made at least one claim per year for IPF. The earliest claim date for IPF was defined as the index date, with incidence calculated based on the year of earliest claim.

### Definitions of comorbidities and other parameters

The demographic characteristics, comorbidities, prescribed pirfenidone, and medical costs of patients with IPF were analysed. Comorbidities were classified as respiratory or non-respiratory. Respiratory comorbidities included COPD, lung cancer, pulmonary embolism, pulmonary hypertension, and obstructive sleep apnoea, whereas non-respiratory comorbidities included GERD, dyslipidaemia, hypertension, diabetes mellitus, ischaemic heart disease, anxiety, depression, and congestive heart failure. The prevalence of comorbidities was assessed at (i) the time of diagnosis, defined as the period from 1 year before to initial diagnosis of IPF; (ii) 1 year after diagnosis, defined as the period from initial diagnosis of IPF to 1 year later; and (iii) 3 years after diagnosis, defined as the period from 1 to 3 years after the initial dignosis of IPF. The occurrence of comorbidities was defined by at least one claim for disease-specific ICD-10 codes (Additional file 1). The objective burden of comorbidities was determined using the Charlson comorbidity index (CCI) [14].

We compared study population by age (< 70 vs. ≥ 70 years), sex, CCI (0–3 vs. ≥ 4) and pirfenidone use, with the latter classified into two groups, those treated with pirfenidone more than 3 months (pirfenidone user group) and those treated for less than 3 months or not treated with pirfenidone (pirfenidone non-user group). In pirfenidone user group, we classified into two groups, those treated with 600 mg oral pirfenidone three times daily for more than 3 months (standard group) and those treated with < 600 mg oral pirfenidone three times daily for more than 3 months (low dose group). Because the cost of pirfenidone has been covered by the NHIS in Korea since October 2015, this classification applied to patients after 2016.

### Statistical analysis

Categorical variables are presented as number and percentages, and continuous variables as means and standard deviations. Annual prevalence was calculated as the number of the prevalent cases per 100,000 people. Annual incidence was calculated as the number of newly diagnosed IPF patients per 100,000 person-years. The denominator of prevalence and incidence was based on the annual population census obtained from the Korean Statistical Information Service [15]. Patients identified in 2011 were excluded in calculations of incidence for clearance period. Trend test of annual incidence was performed by using Poisson regression. Healthcare utilisation patterns, including hospital admission after incident IPF, and total medical costs for 1 year after IPF diagnosis were assessed according to the burden of comorbidities. Differences between groups were calculated using the chi-square test for categorical variables and the Wilcoxon rank sums test for continuous variables. All statistical analyses were performed using the SAS Enterprise Guide software (7.1 version, SAS Institute, Inc., Cary, NC, USA), with differences having two-sided *p* values < 0.05 regarded as statistically significant.

## Results

### Prevalence and incidence of IPF patients

**Fig. 1:**
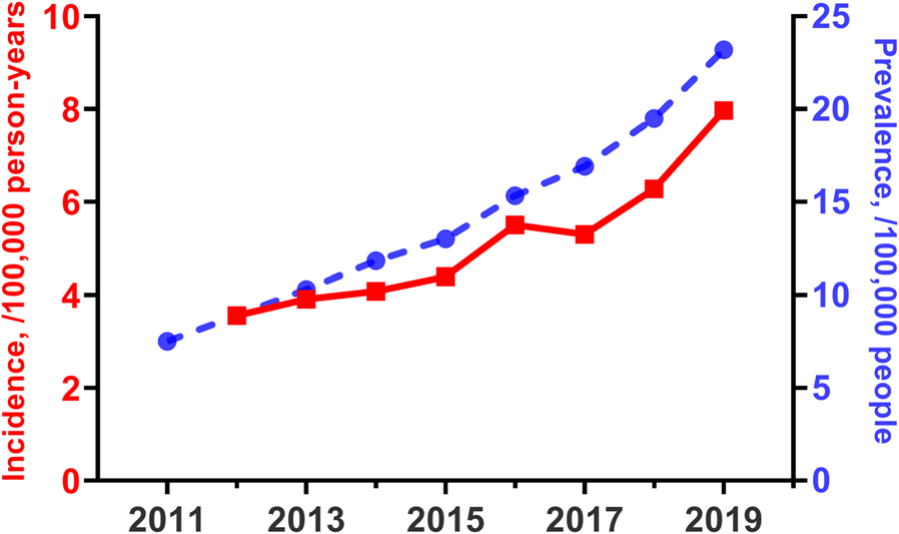
Annual prevalence and incidence rates of idiopathic pulmonary fibrosis from 2011 to 2019 in South Korea. The red solid line shows the incidence/100,000 person-years (left y-axis) and the blue dotted line shows the prevalence/100,000 people (right y-axis). Both prevalence and incidence rates showed statistically significant annual increases (*p* < 0.001 by Poisson regression analysis)

The demographic characteristics of patients with IPF are shown in Table 1. The prevalence and incidence rates of IPF since 2011 were more than twice as high in men as in women. Most IPF patients were included in their seventies, followed by patients in their sixties and eighties. The annual prevalence rate increased from 7.50 to 23.20 per 100,000 people and the annual incidence rate increased from 3.56 to 7.91 per 100,000 person-years in the Korean population (Fig. 1).

**Table 1:** Prevalence and incidence of idiopathic pulmonary fibrosis from 2011 to 2019

	2011	2012	2013	2014	2015	2016	2017	2018	2019
Prevalence study									
Patients, n	3804	4527	5270	6066	6704	7927	8763	10,104	12,031
Sex									
Male	2568	3083	3629	4160	4627	5524	6208	7251	8787
Female	1236	1444	1641	1906	2077	2403	2555	2853	3244
Age, years									
≤ 29	13	11	14	17	15	17	14	15	17
30–39	22	28	33	29	30	31	24	25	23
40–49	88	114	111	115	122	132	125	126	118
50–59	516	540	645	719	667	694	723	786	766
60–69	1229	1439	1593	1765	1934	2375	2586	2912	3473
70–79	1509	1853	2203	2546	2856	3330	3693	4288	5094
≥ 80	427	542	671	875	1080	1348	1598	1952	2540
Prevalence,/100,000 people									
Total	7.50	8.89	10.30	11.82	13.01	15.33	16.92	19.50	23.20
Men	10.11	12.09	14.18	16.21	17.96	21.39	24.01	28.03	33.97
Women	4.88	5.68	6.42	7.43	8.06	9.29	9.86	10.99	12.48
Incidence study									
Patients, n		1812	2001	2094	2261	2843	2743	3255	4099
Sex									
Male		1258	1384	1417	1548	2013	1977	2378	3054
Female		554	617	677	713	830	769	877	1045
Age, years									
≤ 29		7	5	5	6	4	3	3	5
30–39		10	16	10	13	10	4	10	8
40–49		51	37	45	44	42	39	42	40
50–59		193	250	243	211	237	247	252	240
60–69		564	563	581	604	857	776	926	1146
70–79		754	837	869	979	1170	1133	1359	1724
≥ 80		233	293	341	404	523	544	663	936
Incidence/100,000 person-years									
Total		3.56	3.91	4.08	4.39	5.50	5.30	6.28	7.91
Men		4.93	5.41	5.52	6.01	7.79	7.65	9.19	11.81
Women		2.18	2.41	2.64	2.77	3.21	2.97	3.38	4.02

Prevalence and incidence rates were calculated based on the annual population census determined by the Korean Statistical Information Service

Poisson regression analysis of linear trend showed that the annual incidences of idiopathic pulmonary fibrosis were higher in the total Korean population, as well as in men and women (*p* < 0.001 each)

### Prevalence of comorbidities in IPF patients

The prevalence of comorbidities was evaluated in the 21,111 patients with IPF by age and sex (Tables 2 and 3). The most common respiratory comorbidity in all subgroups was COPD (37.34%), followed by lung cancer (3.34%), whereas the most common non-respiratory comorbidities were GERD (70.83%), dyslipidaemia (62.93%) and hypertension (59.04%).

**Table 2:** Baseline characteristics and comorbidities based on sex in patients with idiopathic pulmonary fibrosis

	Total	Men	Women	*p* value
	(n = 21,111)	(n = 15,029)	(n = 6082)	
*Respiratory diseases*				
COPD	7882 (37.34%)	6099 (40.58%)	1783 (29.32%)	< 0.001
Lung cancer	706 (3.34%)	618 (4.11%)	88 (1.45%)	< 0.001
Pulmonary embolism	338 (1.60%)	232 (1.54%)	106 (1.74%)	0.296
Pulmonary hypertension	315 (1.49%)	167 (1.11%)	148 (2.43%)	< 0.001
Obstructive sleep apnoea	65 (0.31%)	55 (0.37%)	10 (0.16%)	0.017
*Non-respiratory diseases*				
GERD	14,953 (70.83%)	10,495 (69.83%)	4458 (73.30%)	< 0.001
Dyslipidaemia	13,286 (62.93%)	9411 (62.62%)	3875 (63.71%)	0.136
Hypertension	12,464 (59.04%)	8817 (58.67%)	3647 (59.96%)	0.083
Diabetes mellitus	9483 (44.92%)	6977 (46.42%)	2506 (41.20%)	< 0.001
Ischaemic heart disease	5431 (25.73%)	4118 (27.40%)	1313 (21.59%)	< 0.001
Anxiety	4238 (20.07%)	2730 (18.16%)	1508 (24.79%)	< 0.001
Depression	3142 (14.88%)	2007 (13.35%)	1135 (18.66%)	< 0.001
Congestive heart failure	2887 (13.68%)	1992 (13.25%)	895 (14.72%)	0.005
*CCI*				0.001
0	577 (2.73%)	423 (2.81%)	154 (2.53%)	
1	3109 (14.73%)	2196 (14.61%)	913 (15.01%)	
2	4109 (19.46%)	2887 (19.21%)	1222 (20.09%)	
3	3823 (18.11%)	2642 (17.58%)	1181 (19.42%)	
≥ 4	9493 (44.97%)	6881 (45.78%)	2612 (42.95%)	
*CCI, mean (standard deviation)*	3.67 (2.40)	3.74 (2.48)	3.51 (2.20)	< 0.001

All values are presented as number (%)

*CCI* Charlson comorbidity index, *COPD* chronic obstructive pulmonary disease, *GERD* gastro-oesophageal reflux disease

*P* values calculated by chi-square tests or Wilcoxon rank sums test

**Table 3:** Baseline characteristics and comorbidities based on age in patients with idiopathic pulmonary fibrosis

	Total (n = 21,111)	Older age (≥ 70 years) (n = 12,762)	Younger age (< 70 years) (n = 8349)	*p* value
*Respiratory diseases*				
COPD	7882 (37.34%)	5027 (39.39%)	2855 (34.20%)	< 0.001
Lung cancer	706 (3.34%)	430 (3.37%)	276 (3.31%)	0.802
Pulmonary embolism	338 (1.60%)	245 (1.92%)	93 (1.11%)	< 0.001
Pulmonary hypertension	315 (1.49%)	175 (1.37%)	140 (1.68%)	0.073
Obstructive sleep apnoea	65 (0.31%)	34 (0.27%)	31 (0.37%)	0.179
*Non-respiratory diseases*				
GERD	14,953 (70.83%)	9065 (71.03%)	5888 (70.52%)	0.427
Dyslipidaemia	13,286 (62.93%)	8289 (64.95%)	4997 (59.85%)	< 0.001
Hypertension	12,464 (59.04%)	8595 (67.35%)	3869 (46.34%)	< 0.001
Diabetes mellitus	9483 (44.92%)	6245 (48.93%)	3238 (38.78%)	< 0.001
Ischaemic heart disease	5431 (25.73%)	3825 (29.97%)	1606 (19.24%)	< 0.001
Anxiety	4238 (20.07%)	2915 (22.84%)	1323 (15.85%)	< 0.001
Depression	3142 (14.88%)	2222 (17.41%)	920 (11.02%)	< 0.001
Congestive heart failure	2887 (13.68%)	2152 (16.86%)	735 (8.80%)	< 0.001
*CCI*				< 0.001
0	577 (2.73%)	230 (1.80%)	347 (4.16%)	
1	3109 (14.73%)	1532 (12.00%)	1577 (18.89%)	
2	4109 (19.46%)	2250 (17.63%)	1859 (22.27%)	
3	3823 (18.11%)	2287 (17.92%)	1536 (18.40%)	
≥ 4	9493 (44.97%)	6463 (50.64%)	3030 (36.29%)	
*CCI, mean (standard deviation)*	3.67 (2.40)	3.99 (2.48)	3.20 (2.20)	< 0.001

All values are presented as number (%)

*CCI* Charlson comorbidity index, *COPD* chronic obstructive pulmonary disease, *GERD* gastro-oesophageal reflux disease

*P* values calculated by chi-square tests or Wilcoxon rank sums test

Classification by sex showed that, of the respiratory comorbidities, COPD (40.58% vs. 29.32%), lung cancer (4.11% vs. 1.45%), and obstructive sleep apnoea (0.37% vs. 0.16%) were more common in men than in women, whereas pulmonary hypertension (2.43% vs. 1.11%) was more prevalent in women than in men. Evaluation of the non-respiratory comorbidities showed that the rates of diabetes mellitus and ischaemic heart disease were higher in men, whereas GERD, anxiety, depression and congestive heart failure were more frequent in the women group. A higher proportion of patients aged ≥ 70 years had COPD (39.39% vs. 34.20%) and pulmonary embolism (1.92% vs. 1.11%) (Table 3). In addition, all investigated non-respiratory comorbidities except GERD were more frequent in patients aged ≥ 70 years than in those aged < 70 years. CCI, an indicator of the burden of comorbidities, was higher in men than in women and was higher in patients aged ≥ 70 years than < 70 years.

**Fig. 2:**
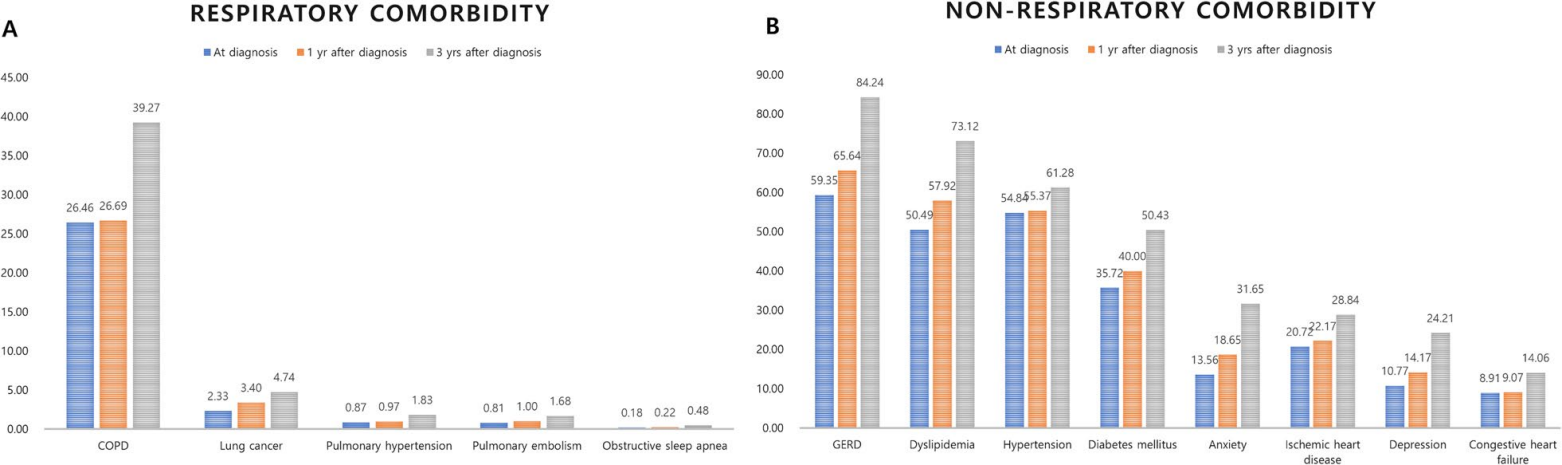
Prevalence of comorbidities in patients with idiopathic pulmonary fibrosis at the time of diagnosis (blue), 1 year after diagnosis (orange) and 3 years after diagnosis (grey). We presented the change of prevalence at three different time points of respiratory comorbidities (**A**) and non-respiratory comorbidities (**B**) Abbreviations: GERD, gastro-oesophageal reflux disease; COPD, chronic obstructive pulmonary disease

The prevalence of comorbidities was evaluated at three different time points, at the time of diagnosis of IPF and 1 and 3 years later (Fig. 2, Additional file 2). Evaluation of the respiratory comorbidities showed that, at these three time points, COPD (26.46%, 26.69%, and 39.27%, respectively) was most common at 3 years after diagnosis, followed by lung cancer (2.33%, 3.40%, and 4.74%, respectively) and pulmonary hypertension (0.87%, 0.97%, and 1.83%, respectively). The most common non-respiratory comorbidity 3 years after diagnosis was GERD, present in most patients with IPF, followed by dyslipidaemia and hypertension. Although the rates of GERD, dyslipidaemia, and hypertension did not markedly differ at the time of initial diagnosis, assessments of their prevalence rates at diagnosis and after 1 and 3 years showed that the rates of GERD (59.35%, 65.64%, and 84.24%, respectively) and dyslipidaemia (50.49%, 57.92%, and 73.12%, respectively) markedly increased over time. The prevalence rates of anxiety and depression were more than twofold higher after 3 years than at the time of diagnosis.

### Demographic characteristics of IPF patients by pirfenidone use

Comparisons of the demographic characteristics and comorbidities in patients who were and were not treated with pirfenidone showed that the proportions of men and of patients aged ≥ 70 years were higher in pirfenidone user groups than in the non-user group (Table 4). Although the proportion of lung cancer was higher in pirfenidone treated patients, the proportion of pulmonary embolism and pulmonary hypertension were higher in untreated patients. Non-respiratory comorbidities, including hypertension, anxiety, depression and congestive heart failure, presented higher proportion in untreated patients, whereas the proportion of GERD and dyslipidaemia were higher in treated patients. The burden of comorbidities was higher in untreated patients than in those who were treated with pirfenidone.

**Table 4:** Clinical characteristics in idiopathic pulmonary fibrosis patients by pirfenidone use since 2016

	Pirfenidone user (n = 5449)	Pirfenidone non-user (n = 7494)	*p* value
*Sex*			< 0.001
Men	4312 (79.13%)	5110 (68.19%)	
Women	1137 (20.87%)	2384 (31.81%)	
*Age*			< 0.001
Older age (≥ 70 years)	2300 (42.21%)	2591 (34.57%)	
Younger age (< 70 years)	3149 (57.79%)	4903 (65.43%)	
*Respiratory diseases*			
COPD	1893 (34.74%)	2671 (35.64%)	0.289
Lung cancer	217 (3.98%)	243 (3.24%)	0.025
Pulmonary embolism	72 (1.32%)	150 (2.00%)	0.003
Pulmonary hypertension	45 (0.83%)	133 (1.77%)	< 0.001
Obstructive sleep apnoea	29 (0.53%)	24 (0.32%)	0.062
*Non-respiratory diseases*			
GERD	4272 (78.40%)	5581 (74.47%)	< 0.001
Dyslipidaemia	3715 (68.18%)	4944 (65.97%)	0.008
Hypertension	3193 (58.60%)	4603 (61.42%)	0.001
Diabetes mellitus	2460 (45.15%)	3495 (46.64%)	0.093
Ischaemic heart disease	1456 (26.72%)	1989 (26.54%)	0.820
Anxiety	934 (17.14%)	1601 (21.36%)	< 0.001
Depression	734 (13.47%)	1228 (16.39%)	< 0.001
Congestive heart failure	726 (13.32%)	1283 (17.12%)	< 0.001
*CCI*			< 0.001
0	170 (3.12%)	178 (2.38%)	
1	826 (15.16%)	966 (12.89%)	
2	1080 (19.82%)	1376 (18.36%)	
3	1030 (18.90%)	1328 (17.72%)	
≥ 4	2343 (43.00%)	3646 (48.65%)	
*CCI, mean (standard deviation)*	2.55 (2.53)	2.51 (2.70)	0.001

All values are presented as number (%)

*CCI* Charlson comorbidity index, *COPD* chronic obstructive pulmonary disease, *GERD* gastro-oesophageal reflux disease

*P* values calculated by chi-square tests or Wilcoxon rank sums test

When classified by dose of pirfenidone, the proportions of men and of patients aged ≥ 70 years were higher in the standard dose than in the low dose group. Except for COPD, there were no differences in proportion of respiratory comorbidities between the standard dose and low dose groups (Additional file 3). The rates of hypertension, anxiety, depression, ischaemic heart disease and congestive heart failure, as well as the burden of comorbidities, were higher in the low dose group.

### Medical resource utilisation by IPF patients

Comparisons of medical resource utilisation in patients with high (CCI ≥ 4) and low (CCI 0–3) burdens of comorbidities within 90 and 365 days showed that, within 90 days, patients with high CCI had utilised more resources associated with admission (Table 5). By contrast, the low burden group had utilised more resources associated with outpatient clinics. Total medical costs were higher at both 90 and 365 days in patients with high than low CCI.

**Table 5:** Charlson comorbidity index stratified medical resource utilisation by patients with idiopathic pulmonary fibrosis

	Within 90 days				Within 365 days			
	Total (n = 21,111)	CCI 0–3 (n = 11,618)	CCI over 4 (n = 9493)	*p* value	Total (n = 21,111)	CCI 0–3 (n = 11,618)	CCI over 4 (n = 9493)	*p* value
Patients who visited outpatient clinics	18,042 (85.46%)	10,395 (89.47%)	7647 (80.55%)	< 0.001	18,170 (86.07%)	10,462 (90.05%)	7708 (81.20%)	< 0.001
Patients admitted to hospitals	4156 (19.69%)	1771 (15.24%)	2385 (25.12%)	< 0.001	5806 (27.50%)	2693 (23.18%)	3113 (32.79%)	< 0.001
Total medical costs (KRW1000)	86.0 [25.0–303.0]	79.2 [24.5–234.0]	97.0 [25.59 –585.7]	< 0.001	314.0 [63.0–1321.0]	303.1 [65.5–961.3]	332.7 [59.7–1963.9]	< 0.001

All values are presented as number (%) or median [interquartile range]

*p* values were calculated using the chi-square test for categorical variables and the Wilcoxon rank sum test for continuous variables

## Discussion

The present utilised Korean nationwide data to evaluate the epidemiology of IPF and its associated comorbidities, and to compare the proportion of these comorbidities in groups classified by sex, age and pirfenidone use. This study found that both the prevalence and incidence of IPF in South Korea increased annually over time. In addition, the proportion rates of comorbidities differed among groups, with specific comorbidities being associated with pirfenidone use. The prevalence of comorbidities increased over time, with an increased burden of comorbidities correlating with greater usage of medical resources, including increased rates of hospitalisations and increased medical costs.

The prevalence and incidence of IPF differ by race, nationality and ethnicity, although some of these differences may depend on the definition of IPF [16, 17]. Geographic variabilities in the incidence and prevalence of IPF have also been reported, but these associations remain unclear [2, 16, 17]. The incidence of IPF was recently reported to be higher in Asia–Pacific countries than in other countries, with the incidence being especially high in South Korea [2]. Because several of the diagnostic criteria of IPF have changed and accurate diagnoses are difficult, even according to guidelines, a working diagnosis is important [1, 18]. In fact, there are disagreements of diagnosis for IPF among physicians in real-world practice. Moreover, differences among studies in the incidence and prevalence of IPF may be due to differences in operational definitions and age inclusion criteria [2, 16, 17, 19]. Because patients with IPF were defined according to combinations of ICD-10 and RID codes, thus clarifying its diagnosis, the absolute number of patients included in the present study was lower than that in a previous study in the Korean population [19]. Further large studies with common criteria for enrolment are needed to accurately determine the prevalence and incidence of IPF, as well as differences among countries.

This study found that COPD (37.34%) was the most common respiratory comorbidity, followed by lung cancer (3.34%) and pulmonary embolism (1.60%), whereas the most common non-respiratory comorbidities were GERD (70.83%), dyslipidaemia (62.93%), and hypertension (59.04%). The prevalence rates of these comorbidities were comparable to those in previous studies, although the reported ranges of comorbidity rates were very wide [8, 20]. When compared to general population in South Korea, it was investigated that the prevalence rate of the comorbidities described above was higher in patients with IPF (COPD: 13.4%, lung cancer: 0.2%, pulmonary embolism: less than 0.1%, GERD: 6.4%, dyslipidaemia: 20.7%, and hypertension: 32.0% in general population in South Korea) [21–26]. Because most of IPF patients have abnormal pulmonary function test results with restrictive pattern, it was difficult to find and diagnose combined COPD in real practice. Although previous study presented the prevalence of COPD in IPF patients was 47.6% in USA and 39.8% in Germany, several retrospective cohort studies in South Korea reported lower prevalence, about 3 to 5%, of COPD in IPF patients [27–30]. Therefore, further prospective, and large study with concise pulmonary function test might be needed to clarify this point. Compared with other studies, the prevalence of obstructive sleep apnoea was exceptionally low [14, 31, 32], with one study reporting that its prevalence was 4.5% in men and 3.2% in women [33]. The lower prevalence of obstructive sleep apnoea in the Korean general population than in western countries may be due to Asian populations having a lower rate of obesity, which is regarded as one of the most important risk factors for obstructive sleep apnoea [33]. A single-centre retrospective study found that 37 (65%) of 57 South Korean patients with IPF who underwent polysomnography were diagnosed with obstructive sleep apnoea, suggesting that the percentage of IPF patients with obstructive sleep apnoea may be underestimated in the absence of polysomnography [34]. This study also found that most comorbidities were more common in IPF patients aged ≥ 70 years than in those aged < 70 years, in agreement with results showing that comorbidity rates were higher in IPF patients aged ≥ 75 years than in younger IPF patients [31]. Because the ageing process influences the pathogenesis of IPF, comorbidities must be carefully monitored and managed in patients with IPF, especially in older patients [35, 36].

Of the 12,943 IPF patients since 2016 in this study, 5449 (42.10%) had been treated with pirfenidone. This relatively low rate of pirfenidone use may be due to the lack of insurance coverage for this medication prior to October 2015 and/or to our definition of pirfenidone use as treatment with this agent for more than 3 months. A recent real-world cohort study of a Korean population found that only 656 (48.77%) of 1345 patients with IPF had been treated with antifibrotic agents, including pirfenidone and nintedanib [37]. This low rate indicates a need to encourage clinicians in South Korea to prescribe antifibrotic agents for IPF. Although causal relationships could not be determined, the proportion of several comorbidities differed in patients who were and were not treated with pirfenidone. Patients with comorbidities and high CCI, especially a CCI ≥ 4, might have lower rate of use of pirfenidone due to many medications taken together and general weakness [38]. In particular, the proportion of anxiety and depression were higher in patients in pirfenidone non-user group. Patients with IPF were shown to be more vulnerable to psychological problems than the general population [8]. Severe dyspnoea, decreased quality of life, and greater requirements for social support are regarded as psychological distress in IPF patients [39, 40]. Reduced drug adherence has been observed in patients with other diseases who experience depression or anxiety [41, 42]. Although the prevalence rates of both psychiatric diseases were reported lower in the present study than in other studies using the diagnostic tools to confirm depression and anxiety, the lower rate in the present study may have been caused by the underdiagnosis of psychiatric symptoms [13, 20]. By contrast, the present study found that the proportion of lung cancer was higher in pirfenidone user group. Because previous studies had suggested that pirfenidone might reduce some of the adverse effects associated with agents used to treat lung cancer, physicians may be more likely to prescribe pirfenidone to IPF patients with lung cancer [43, 44]. Additionally, there may be several other reasons. At first, closer monitoring for patients, who received the antifibrotics, could increase rate of detected lung cancer. Secondly, before 2019, pirfenidone were prescribed for IPF patients, who a range of 50% or more of the predicted FVC, 35% or more of the predicted carbon monoxide diffusing capacity and 150 m or more of distance of six-minute walk test. The range presented the mild to moderate severity of IPF. Severe IPF patients have relatively shorter survival duration and were difficult to take examination for diagnosis of lung cancer, these facts could underestimate the proportion of combined lung cancer in IPF patients. Additionally, mild IPF patients might have relatively shorter disease duration or lower disease activity. As we presented in Fig. 2A, the proportion of lung cancer was increased as time progressed in IPF patients. Finally, older and more male patients were included in the pirfenidone user group. Old age and male sex were well-known risk factor of lung cancer in general population. Although the effect size might be relatively small, these factors could affect the proportion of lung cancer.

Several studies have evaluated the relationships between comorbidities and clinical outcomes in patients with IPF [8, 9, 20]. For example, cardiac disease and lung cancer were significant predictors of mortality, whereas GERD and diastolic dysfunction improved survival [10]. Moreover, both the presence and treatment of comorbidities was found to affect prognosis in patients with interstitial lung disease [45]. In our study, the prevalence of most comorbidities was higher 3 years after diagnosis than at the time of diagnosis, although the rates of increase varied among these comorbidities. More than one-third of IPF patients were affected by six comorbidities. The prevalence of several comorbidities increased more than twofold, except for ischaemic heart disease and congestive heart failure. A CCI ≥ 4 was associated with increased rates of hospitalisations and increased total medical costs, suggesting that comorbidities might affect the clinical course of IPF. Similarly, comorbidities in patients with interstitial lung disease increased hospitalisations, but may be improved with proper management [46].

The present study had several limitations. First, IPF was defined based on the ICD-10 code at the time of diagnosis. Although actual diagnosis was associated with the operational definition based on ICD-10 code, our use of the latter may overestimate or underestimate the prevalence and incidence rates of IPF and comorbidities [47]. To improve the accuracy of the operational diagnosis of IPF, we combined the ICD-10 code and RID code. Many studies using operational definition with RID code presented more than 90% of sensitivity or specificity in various diseases [47–49]. However, because operational definition of IPF with RID code were not verified, further studies to validate IPF definition using RID code by comparison with other well established and validated operational definition [50]. Second, we were able to assess a limited number of baseline characteristics because the HIRA database includes limited personal information due to the Korean Personal Information Protection Act. Similarly, we could not analyse mortality as a study outcome because information about patient death was unavailable. Third, because nintedanib was not covered by insurance in South Korea, the only antifibrotic agent included in this study was pirfenidone. Likewise, because only patients diagnosed with IPF were included in this study, this result could not be applied to patients with progressive pulmonary fibrosis with other ILD subtypes. Because recently published studies suggested operational definition of progressive pulmonary fibrosis with other ILD subtypes, it can be possible to investigate the prevalence of comorbidities in progressive pulmonary fibrosis patients with other ILD subtypes by using nationwide claim data [51, 52]. Finally, because this study was a nationwide population-based study based on health claims data, we could only determine the associations between variables, rather than causal relationships. Additionally, we only investigated existence of comorbidities based on the health claim data and could not validate comorbidities. This method could be affected by misdiagnosis and lead to overestimation for prevalence of comorbidities. Therefore, further prospective, including other antifibrotic agents and other ILD subtypes, might be required to overcome these limitations.

## Conclusions

In conclusion, this study found that the prevalence and incidence rates of IPF in the Korean population increased annually. Many patients with IPF had respiratory and non-respiratory comorbidities, although the prevalence was dependent on sex, age and use of pirfenidone. The prevalence of these comorbidities was higher 3 years after than at the time of IPF diagnosis and affected the use of pirfenidone and medical resources. Additional studies are needed to clarify causal relationships.

## Supplementary Information

**Additional file 1**. International Classification of Diseases, 10th revision codes (ICD-10 codes) for the diseases evaluated in this study.

**Additional file 2**. Prevalence of comorbidities in patients with idiopathic pulmonary fibrosis during the 3 years after initial diagnosis.

**Additional file 3**. Clinical characteristics of patients with idiopathic pulmonary fibrosis based on the pirfenidone dose since 2016.

## Data Availability

The data that support the findings of this study are available from National Health Insurance Sharing Service in Korea, but restrictions apply to the availability of these data, which were used under license for the current study, and so are not publicly available. Data are however available from the authors upon reasonable request and with permission of National Health Insurance Sharing Service in Korea.

## Abbreviations

CCI: Charlson comorbidity index
COPD: Chronic obstructive pulmonary disease
GERD: Gastro-oesophageal reflux disease
HIRA: Health Insurance Review and Assessment
ICD-10: The 10th revision of the International Classification of Disease and Related Health Problems
IPF: Idiopathic pulmonary fibrosis
NHIS: National Health Insurance System
RID: Rare Intractable Diseases
