# Supplementary material for: Epidemiology and comorbidities in idiopathic pulmonary fibrosis: a nationwide cohort study
Source: BMC Pulm Med. 2023 Feb 4;23:54. doi: 10.1186/s12890-023-02340-8 (PMC9898951; doi:10.1186/s12890-023-02340-8)
Supplement: Supplementary file 1 — Additional file 1. International Classification of Diseases, 10th revision codes (ICD-10 codes) for the diseases evaluated in this study. [file 12890_2023_2340_MOESM1_ESM.docx]

Additional file 1. International Classification of Diseases, 10th revision codes (ICD-10 codes) for the diseases evaluated in this study

| Disease | ICD 10 Codes |
| --- | --- |
| Respiratory diseases  COPD  Lung cancer  Pulmonary embolism  Pulmonary hypertension  Obstructive sleep apnoea | J44  C34  I26  I27.0, 27.2, 27.8, 27.9  G47.30 |
| Non respiratory diseases  GERD  Dyslipidaemia  Hypertension  Diabetes mellitus  Ischaemic heart disease  Anxiety  Depression  Congestive heart failure | K21  E78  I10-13, 15  E10-14  I20-25  F40, 41  F32, 33  I50 |

Abbreviation: COPD, chronic obstructive pulmonary disease; GERD, gastro-oesophageal reflux disease.
