# Supplementary material for: Epidemiology and comorbidities in idiopathic pulmonary fibrosis: a nationwide cohort study
Source: BMC Pulm Med. 2023 Feb 4;23:54. doi: 10.1186/s12890-023-02340-8 (PMC9898951; doi:10.1186/s12890-023-02340-8)
Supplement: Supplementary file 2 — Additional file 2. Prevalence of comorbidities in patients with idiopathic pulmonary fibrosis during the 3 years after initial diagnosis. [file 12890_2023_2340_MOESM2_ESM.docx]

Additional file 2. Prevalence of comorbidities in patients with idiopathic pulmonary fibrosis during the 3 years after initial diagnosis.

|  | At diagnosis  (n = 21,111) | 1 yr after diagnosis  (n = 12,155) | 3 yrs after diagnosis  (n = 5,406) |
| --- | --- | --- | --- |
| Respiratory diseases  COPD  Lung cancer  Pulmonary hypertension  Pulmonary embolism  Obstructive sleep apnoea | 5,586 (26.46%)  492 (2.33%)  184 (0.87%)  172 (0.81%)  39 (0.18%) | 3,244 (26.69%)  413 (3.40%)  118 (0.97%)  122 (1.00%)  27 (0.22%) | 2,123 (39.27%)  256 (4.74%)  99 (1.83%)  91 (1.68%)  26 (0.48%) |
| Non respiratory diseases  GERD  Dyslipidaemia  Hypertension  Diabetes mellitus  Anxiety  Ischaemic heart disease  Depression  Congestive heart failure | 12,530 (59.35%)  10,658 (50.49%)  11,577 (54.84%)  7,540 (35.72%)  2,862 (13.56%)  4,375 (20.72%)  2,273 (10.77%)  1,881 (8.91%) | 7,979 (65.64%)  7,040 (57.92%)  6,730 (55.37%)  4,862 (40.00%)  2,267 (18.65%)  2,695 (22.17%)  1,722 (14.17%)  1,103 (9.07%) | 4,554 (84.24%)  3,953 (73.12%)  3,313 (61.28%)  2,726 (50.43%)  1,711 (31.65%)  1,559 (28.84%)  1,309 (24.21%)  760 (14.06%) |
| CCI  0  1  2  3  ≥ 4 | 577 (2.73%)  3,109 (14.73%)  4,109 (19.46%)  3,823 (18.11%)  9,493 (44.97%) | 812 (6.68%)  2,226 (18.31%)  2,560 (21.06%)  2,086 (17.16%)  4,471 (36.78%) | 102 (1.89%)  469 (8.68%)  794 (14.69%)  880 (16.28%)  3,161 (58.47%) |
| CCI, mean (standard deviation) | 3.67 (2.40) | 3.21 (2.40) | 4.55 (2.82) |

All values are presented as number (%).

Abbreviations: CCI, Charlson comorbidity index; COPD, chronic obstructive pulmonary disease; GERD, gastro-oesophageal reflux disease
