# Supplementary material for: Epidemiology and comorbidities in idiopathic pulmonary fibrosis: a nationwide cohort study
Source: BMC Pulm Med. 2023 Feb 4;23:54. doi: 10.1186/s12890-023-02340-8 (PMC9898951; doi:10.1186/s12890-023-02340-8)
Supplement: Supplementary file 3 — Additional file 3. Clinical characteristics of patients with idiopathic pulmonary fibrosis based on the pirfenidone dose since 2016. [file 12890_2023_2340_MOESM3_ESM.docx]

Additional file 3. Clinical characteristics of patients with idiopathic pulmonary fibrosis based on the pirfenidone dose since 2016

|  | Pirfenidone  standard dose  (n = 1,934) | Pirfenidone  low dose  (n = 3,515) | *P* value |
| --- | --- | --- | --- |
| Sex  Men  Women | 1,663 (85.99%)  271 (14.01%) | 2,649 (75.36%)  866 (24.64%) | < 0.001 |
| Age  Older age (≥70 years)  Younger age (<70 years) | 1,041 (53.83%)  893 (46.17%) | 1,259 (35.82%)  2,256 (64.18%) | < 0.001 |
| Respiratory diseases  COPD  Lung cancer  Pulmonary embolism  Pulmonary hypertension  Obstructive sleep apnoea | 609 (31.49%)  90 (4.65%)  22 (1.14%)  12 (0.62%)  13 (0.67%) | 1,284 (36.53%)  127 (3.61%)  50 (1.42%)  33 (0.94%)  16 (0.46%) | 0.001  0.060  0.378  0.214  0.292 |
| Non respiratory diseases  GERD  Dyslipidaemia  Hypertension  Diabetes mellitus  Ischaemic heart disease  Anxiety  Depression  Congestive heart failure | 1,528 (79.01%)  1,308 (67.63%)  1,057 (54.65%)  851 (44.00%)  464 (23.99%)  271 (14.01%)  218 (11.27%)  209 (10.81%) | 2,744 (78.07%)  2,407 (68.48%)  2,136 (60.77%)  1,609 (45.78%)  992 (28.22%)  663 (18.86%)  516 (14.68%)  517 (14.71%) | 0.419  0.521  <0.001  0.208  0.001  <0.001  <0.001  <0.001 |
| CCI  0  1  2  3  ≥ 4 | 72 (3.72%)  303 (15.67%)  418 (21.61%)  373 (19.29%)  768 (39.71%) | 98 (2.79%)  523 (14.88%)  662 (18.83%)  657 (18.69%)  1,575 (44.81%) | <0.002 |
| CCI, mean (standard deviation) | 2.57 (2.47) | 2.53 (2.56) | 0.170 |

All values are presented as number (%).

The *P* values were calculated using the chi-square test or Wilcoxon rank sums test.

Abbreviation: CCI, Charlson comorbidity index; COPD, chronic obstructive pulmonary disease; GERD, gastro-oesophageal reflux disease
